# Radiomics-based classification of hepatocellular carcinoma and hepatic haemangioma on precontrast magnetic resonance images

**DOI:** 10.1186/s12880-019-0321-9

**Published:** 2019-03-11

**Authors:** Jingjun Wu, Ailian Liu, Jingjing Cui, Anliang Chen, Qingwei Song, Lizhi Xie

**Affiliations:** 1 Department of Radiology, the First Affiliated Hospital of Dalian Medical University; Xigang district, Zhongshan road, No.222, Dalian, China; 2 Huiying Medical Technology Co., Ltd, Beijing, China; 3 GE Healthcare, MR Research, Beijing, China

**Keywords:** Radiomics, Hepatocellular carcinoma, Hepatic haemangioma, Magnetic resonance imaging, Classification

## Abstract

**Background:**

To evaluate the feasibility of using radiomics with precontrast magnetic resonance imaging for classifying hepatocellular carcinoma (HCC) and hepatic haemangioma (HH).

**Methods:**

This study enrolled 369 consecutive patients with 446 lesions (a total of 222 HCCs and 224 HHs). A training set was constituted by randomly selecting 80% of the samples and the remaining samples were used to test. On magnetic resonance (MR) images of HCC and HH obtained with in-phase, out-phase, T2-weighted imaging (T2WI), and diffusion-weighted imaging (DWI) sequences, we outlined the target lesions and extracted 1029 radiomics features, which were classified as first-, second-, higher-order statistics and shape features. Then, the variance threshold, select k best, and least absolute shrinkage and selection operator algorithms were explored for dimensionality reduction of the features. We used four classifiers (decision tree, random forest, K nearest neighbours, and logistic regression) to identify HCC and HH on the basis of radiomics features. Two abdominal radiologists also performed the conventional qualitative analysis for classification of HCC and HH. Diagnostic performances of radiomics and radiologists were evaluated by receiver operating characteristic (ROC) analysis.

**Results:**

Valuable radiomics features for building a radiomics signature were extracted from in-phase (*n* = 22), out-phase (*n* = 24), T2WI (*n* = 34) and DWI (n = 24) sequences. In comparison, the logistic regression classifier showed better predictive ability by combining four sequences. In the training set, the area under the ROC curve (AUC) was 0.86 (sensitivity: 0.76; specificity: 0.78), and in the testing set, the AUC was 0.89 (sensitivity: 0.822; specificity: 0.714). The diagnostic performance for the optimal radiomics-based combined model was significantly higher than that for the less experienced radiologist (2-years experience) (AUC = 0.702, *p* < 0.05), and had no statistic difference with the experienced radiologist (10-years experience) (AUC = 0.908, p>0.05).

**Conclusions:**

We developed and validated a radiomics signature as an adjunct tool to distinguish HCC and HH by combining in-phase, out-phase, T2W, and DW MR images, which outperformed the less experienced radiologist (2-years experience), and was nearly equal to the experienced radiologist (10-years experience).

## Background

Hepatocellular carcinoma (HCC) and hepatic haemangioma (HH) are common hepatic malignant and benign tumours respectively [1, 2]. Early detection and diagnosis of these tumours are extremely important because their treatment strategies and prognoses are completely different [3]. However, HCC and HH share similar signatures in the early stages, when the “gold standard” for differential diagnosis mainly involves pathological evidence obtained post-surgery, puncture, or interventional tumour cell staining [4]. Magnetic resonance imaging (MRI) has recently gained prominence in the diagnosis of hepatic tumours because of its various modalities such as multi-parametric imaging, functional imaging, and biochemical metabolic analysis techniques, all of which can optimize the clinical application from morphology to quantitative analysis [5, 6]. The differential diagnosis of HCC and HH by medical images usually depends on the visually morphological features and signal intensity, and the changes of signal enhancement such as washout is currently the key evidence for imaging diagnosis. However, diagnosis in patients who cannot receive contrast injections may be problematic because non-contrast image features have inferior diagnosis accuracy. Moreover, the absence of washout in small HCCs may also lead to incorrect differential diagnosis [7]. It is note worthy that currently diagnostic methods based on MR images may be influenced by human factors, such as tiredness or inexperience, which can potentially affect the diagnostic accuracy. These reasons highlight the need for a quantitative diagnostic criterion based on precontrast MR images that does not require invasive approaches, such as surgery, puncture, or interventional therapy, or intravenous injection of a contrast agent.

Radiomics is a diagnostic technology based on radiomics signatures that is currently gaining prominence in the field of radiology for its potential ability to help detect lesions, improve diagnostic accuracy, predict disease risk, and guide treatment strategies [8]. Preclinical studies of radiomics-based approaches with imaging methods such as X-ray radiography, computed tomography (CT), ultrasound, and MRI have been performed for various tissues such as the lung, liver, bone, and brain [9–11]. For MR images of liver lesions, Kim et al. [12] suggested that radiomics was useful in grading HCC risk and showed consistency with radiologists’ opinions. Gatos et al. [13] applied radiomics to segment and classify focal liver lesions on the basis of non-enhanced T2-weighted images, providing a noninvasive method to evaluate liver lesions. This study aimed to determine whether a radiomics signature with high specificity and sensitivity can distinguish between HCC and HH by using precontrast MR images with a radiomics model based on comparisons of four common classifiers.

## Methods

### Dataset

This study was approved by the institutional review board of our hospital, and informed consent was obtained from all patients. In this study, we recruited 369 patients with 446 lesions including HCC (222/446) and HH (224/446) confirmed by pathological examinations after hepatectomy, puncture assessments, interventional tumour cell staining or typical imaging findings between January 1, 2011 and September 1, 2017. A training set was constituted by randomly selecting 80% of the samples and the remaining samples were used to test. All patients had undergone MRI examinations before therapeutic procedures such as surgery, puncture, and adjuvant therapy. MRI was performed using the GE 3 .0T or 1 .5T MR scanner (Signa, HDxt, GE Healthcare, United States) with an eight-channel phased array body coil. All patients were asked to fast for about four to six hours before scanning, after which they underwent upper abdomen MRI examination in the supine position. The MR scan sequences were as follows: (1) fast spoiled gradient-recalled sequence axial fat suppression T1-weighted gradient echo in-phase and out-phase: repetition time (TR)/echo time (TE) = 400/8.0 ms, field of view (FOV) = 320 mm × 320 mm, matrix = 320 × 192, number of excitations (NEX) = 2.0, slice thickness = 5.0 mm. (2) T2WI: TR/TE = 4000/125 ms, FOV = 320 mm × 320 mm, matrix = 320 × 192, NEX = 4.0, slice thickness = 5 .0mm. (3) DWI with a spin-echo planar imaging sequence: TR/TE, 4000/70 ms, b = 600 s/mm [2], slice thickness = 5.0 mm, FOV = 320 mm × 320 mm, matrix = 128 × 128, NEX = 6.0. The patients’ status of hepatic cirrhosis were recorded according to MRI features of cirrhosis: a nodular liver margin, lobar atrophy / hypertrophy, parenchymal heterogeneity. Finally, 59 HCC lesions and 19 HH lesions were accompanied by the occurance of cirrhosis.

### Radiomics analysis overview

**Fig. 1:**
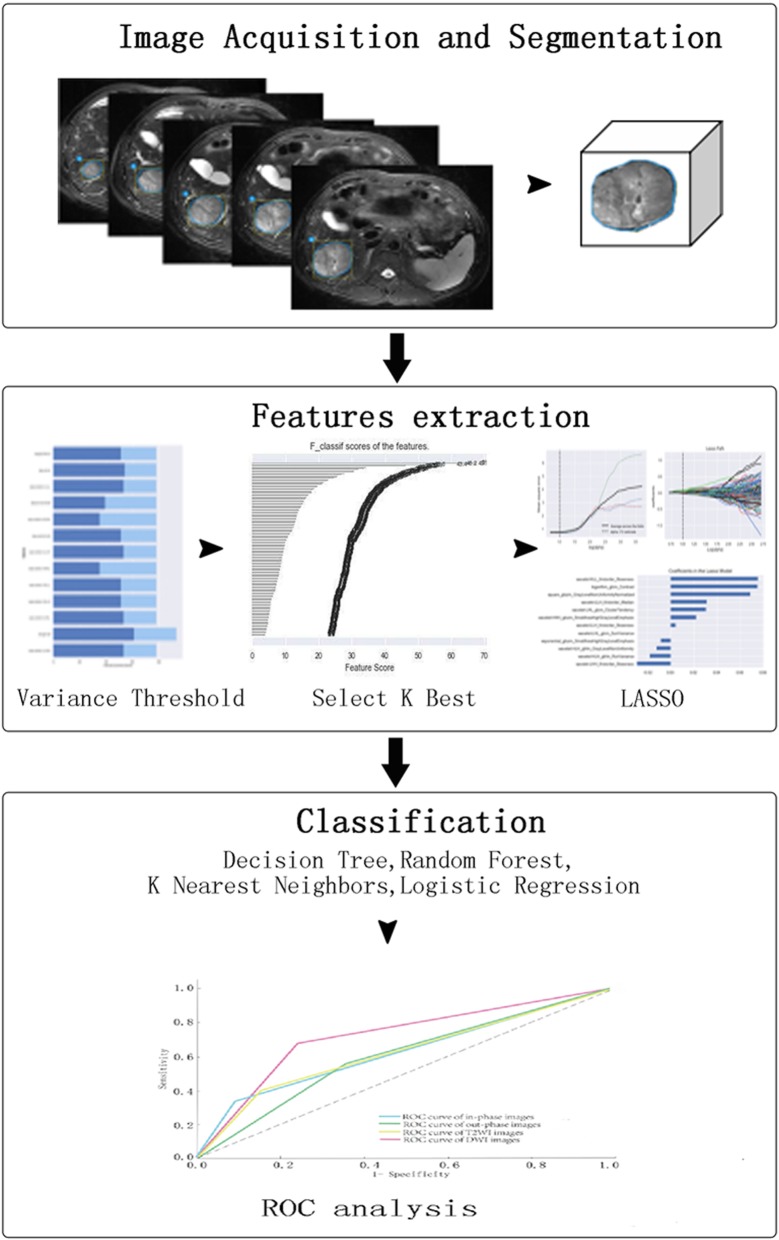
Flow diagram of theradiomics analysis

In the abdominal MR images on each sequence, including in-phase T1WI, out-phase T1WI, T2WI, and DWI, the lesions at all slices were outlined as regions of interest (ROIs), which were then reconstituted to form stereoscopic lesions. In addition, we extracted 1029 radiomics features, including original (*n* = 93) and higher-order features (*n* = 936). Next, the variance threshold, select k best and least absolute shrinkage and selection operator (LASSO) operator algorithms were recommended to reduce the dimensionality of the radiomics features. Four classifiers (decision tree, random forest, K nearest neighbours, and logistic regression) were used to distinguish HCC and HH. The diagnostic performance was evaluated on the basis of the area under the receiver operating characteristic curve (AUC), sensitivity and specificity. Figure 1 illustrates the overall flowchart of our radiomics workflow.

### Imaging segmentation

**Fig. 2:**
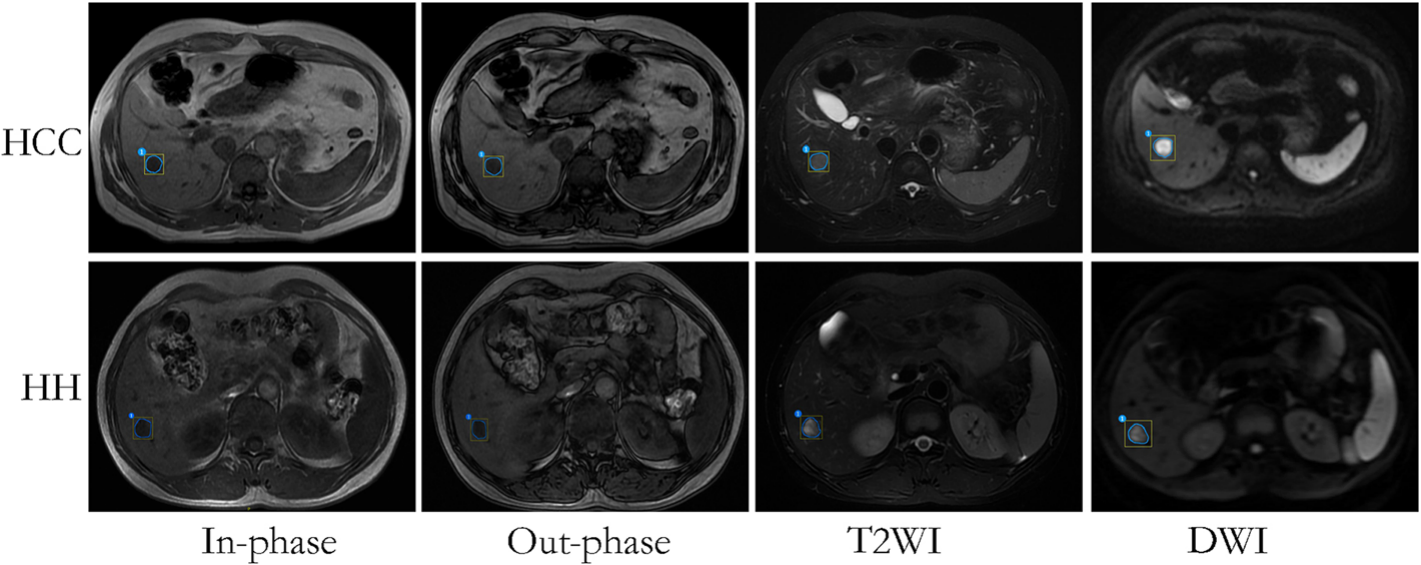
Imaging segmentation on HCC and HH. The ROIs which enclose the boundary of target lesions on in-phase, out-phase, T2WI and DWI are depicted

On the basis of the abdominal MRI data from the Picture Archiving and Communication System, in-phase T1WI, out-phase T1WI, T2WI, and DWI were collected. We manually outlined the lesions and attempted error minimization. Two radiologists with five years’ clinical experience were blinded to all patients’ information, reviewed the selected MR images, and outlined target lesions on each image. Consistency in their ROI assessments was regarded as a sign of validity. However, if they had different opinions, a third radiologist with ten years’ clinical experience participated in the discussion and the final decision were recorded. The examples of ROIs in HCC and HH in different MRI series including in-phase imaging, out-phase imaging, T2WI, and DWI are shown in Fig. 2.

### Intensity normalization

Even with the same MRI scanner and identical scanning parameters, variations in the MRI intensity cannot be avoided, and these may affect the process of feature extraction. To minimize the MRI intensity variations, we normalized the intensity of the image using the following formula (where *x* indicates the original intensity; *f(x)* indicates the normalized intensity; *μ* refers to the mean value; *σ* indicates the variance; *s* is an optional scaling, by default, it is set to 1) [14].

$$ \mathrm{f}(x)=\frac{s\left(x-{\mu}_x\right)}{\delta_x} $$

### Radiomics feature extraction

We analysed a total of 1029 radiomics features categorized into four classes: first-order features, shape features, second-order features (texture), and higher-order statistic features (Table 1). First-order features can provide the spatial distribution of multiple voxel intensities regardless of the three-dimensional structure. Based on the histogram method within the image region, it is feasible to analyse the image features via voxel distribution. Shape features of tumours, such as oval, spiculated, lobulated, and irregular, which are hypothesized as potential identification biomarkers, are independent from the grey-level intensity distribution in the ROIs. We epitomized the shape-related features from a three-dimensional perspective. Considering the sphere as a standard shape, we aimed to analyse the similarity between the lesions and the sphere. The diameter, area, and volume were also taken into account using parameters such as the *maximum 2D diameter column/row/slice, maximum 3D diameter*, and *surface volume ratio*. We also suggested that *elongation* and *flatness* may be potentially shape-related markers. The values of *elongation* range between 1 (where the cross-section through the first and second largest principal moments is circle-like) and 0 (where the object is a single point or a 1-dimensional line). The values of *flatness* also range between 1 (non-flat, sphere-like) and 0 (a flat object).

**Table 1:** Radiomics features in the radiomics analysis

Types		Features
First-order statistics (*n* = 19)		Energy, Total Energy, Entropy, Minimum, 10Percentile, 90Percentile, Maximum, Mean, Median, Interquartile Range, Range, Mean Absolute Deviation, Robust Mean Absolute Deviation, Root Mean Squared, Standard Deviation, Skewness, Kurtosis, Variance, Uniformity
Shape (*n* = 15)		Volume, Surface Area, Surface Volume Ratio, Sphericity, Compactness1, Compactness2, Spherical Disproportion, Maximum 3D Diameter, Maximum 2D Diameter Column, Maximum 2D Diameter Row, Major Axis, Minor Axis, Least Axis, Elongation, Flatness
Second-order statistics	GLCM (*n* = 27)	Autocorrelation, Average Intensity, GrayLevel Intensity, Cluster Prominence, Cluster Shade, Cluster Tendency, Contrast, Correlation, Difference Average, Difference Entropy, Difference Variance, Dissimilarity, Energy, Entropy, Homogeneity1, Homogeneity2, Informal Measure Of Correlation1, Informal Measure Of Correlation2, Inverse Difference Moment, Inverse Difference Moment Normalized, Inverse Difference, Inverse Variance, Maximum Probability, Sum Average, Sum Entropy, Sum Variance, Sum of Squares
	GLRLM (*n* = 16)	Gray Level Non-Uniformity, Gray Level Non Uniformity Normalized, Gray Level Variance, High Gray Level Run Emphasis, Long Run Emphasis, Long Run High Gray Level Emphasis, Long Run Low Gray Level Emphasis, Low Gray Level Run Emphasis, Short Run Emphasis, Short Run High Gray Level Emphasis, Short Run Low Gray Level Emphasis, Run Entropy, Run Length Non Uniformity, Run Length Non Uniformity Normalized, Run Percentage, Run Variance
	GLSZM (*n* = 16)	Small Area Emphasis, Large Area Emphasis, Gray Level Non-Uniformity, Gray Level Non-Normalized, Size Zone Non-Uniformity, Size Zone Non-Uniformity Normalized, Zone Percentage, Gray Level Variance, Zone Variance, Zone Entropy, Low Gray Level Zone Emphasis, High Gray Level Zone Emphasis, Small Area Low Gray Level Emphasis, Small Area High Gray Level Emphasis, Large Area Low Gray Level Emphasis, Large Area High Gray Level Emphasis
Higher-order statistics (n = 936)		First- and second-order features are transformed by Exponential, Square, Square Root, Logarithm, Wavelet (wavelet-LHL,wavelet-LHH,wavelet-HLL,wavelet-LLH,wavelet-HLH,wavelet-HHH,wavelet-HHL,wavelet-LLL)

Note: GLCM, Gray Level Co-occurence Matrix; GLRLM, Gray Level Run Length Matrix;

GLSZM, Gray Level Size Zone Matrix; L, low; H, high

Second-order statistics are generally named as “texture”, which was first suggested by Haralick and his colleagues in 1973 [15, 16]. The texture features are described via a density histogram and the spatial locations of each pixel are also expressed. Three types of texture features, including Gray Level Co-occurence Matrix (GLCM), Gray Level Run Length Matrix (GLRLM), and Gray Level Size Zone Matrix (GLSZM), were analysed in our study.

On the basis of the first- and second-order features, we applied filter grids on images and obtained robust transformed features, namely higher-order statistic features. Five types of filters were applied: exponential, square, square root, logarithm, and wavelet (which was applied as either a high- (H) or a low-pass (L) filter in each of the three dimensions: wavelet-LHL, wavelet-LHH, wavelet-HLL, wavelet-LLH, wavelet-HLH, wavelet-HHH, wavelet-HHL, and wavelet-LLL).

In present study, we used three methods to gradually select the optimal features. We first applied a variance threshold to reduce the features whose variance was not consistent with the threshold (set as 0.8). Next, based on the select K best findings, we further removed the features that did not show statistical differences (*p* > 0.05). This process can be viewed as a preconditioning of the predictive model. Finally, the LASSO algorithm was performed, the best alpha in each sequence was identified, and coefficients were calculated to obtain the most relevant features.

### Classification

Classification was applied to identify HCC and HH on the basis of various features in multi-sequences. The classifiers we used were constructed by supervised learning, which involves learning from a set of given samples possessing known categories to create a classifier that can correctly classify new objects [17]. Four supervised learning classifiers were tested in our study (decision tree, random forest, K nearest neighbours, and logistic regression). The decision tree algorithm first generates readable rules and decisions using an inductive algorithm, after which new data are analysed by using this decision. Thus, a decision tree is essentially a classification algorithm employing a series of rules to classify data [18]. The random forest technique involves a nonlinear supervised sparse regression–based classifier that contains multiple decision trees, with its output category based on the number of categories exported by the individual trees [19]. The K nearest neighbour is a kind of lazy-learning algorithm that implements learning based on the *k* nearest neighbours of each query point during the classification process [20]. In the process of logical regression, to address a regression or classification problem, a cost function is established and then the optimal model parameters are obtained through optimization and iterative solution [21]. The AUC, sensitivity, and specificity were significant indexes for evaluating the performance in differentiating HCC from HH on non-enhanced MR images.

### Classification by two radiologists

The conventional qualitative analysis for classification of HCC and HH was performed by two abdominal radiologists with different experience (2 and 10 years, respectively). They were blinded to all patients’ information, reviewed the MR images, and recorded their own diagnosis. For evaluation of the performances of the two radiologists in the task of classification, the AUC, sensitivity, and specificity were calculated and compared.

## Results

**Fig. 3:**
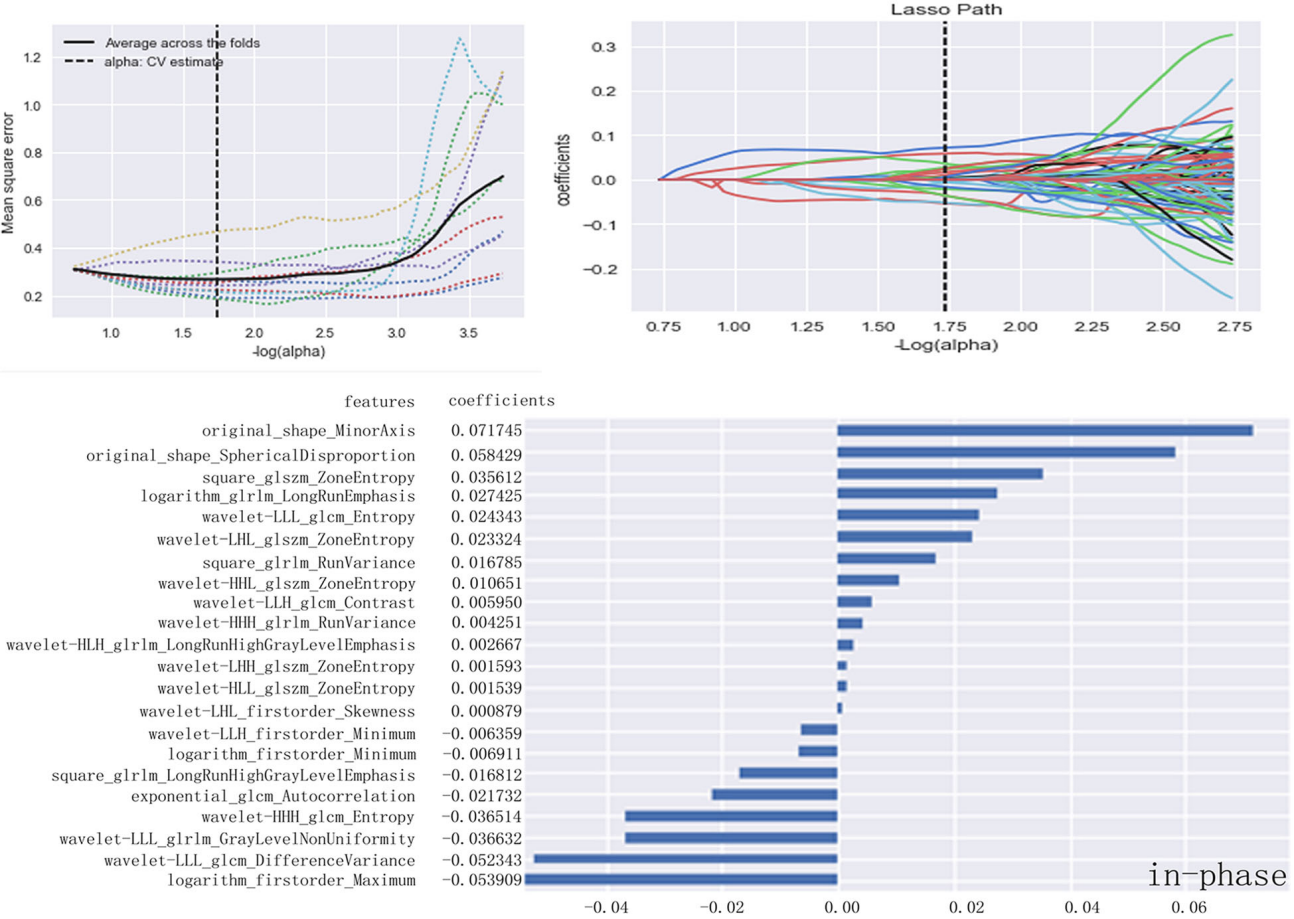
LASSO model on in-phase images. The optimal value of the lasso tuning parameter (alpha = 1.738) is found. And 22 features which are correspond to the optimal alpha value are extracted following coefficients on in-phase images

**Fig. 4:**
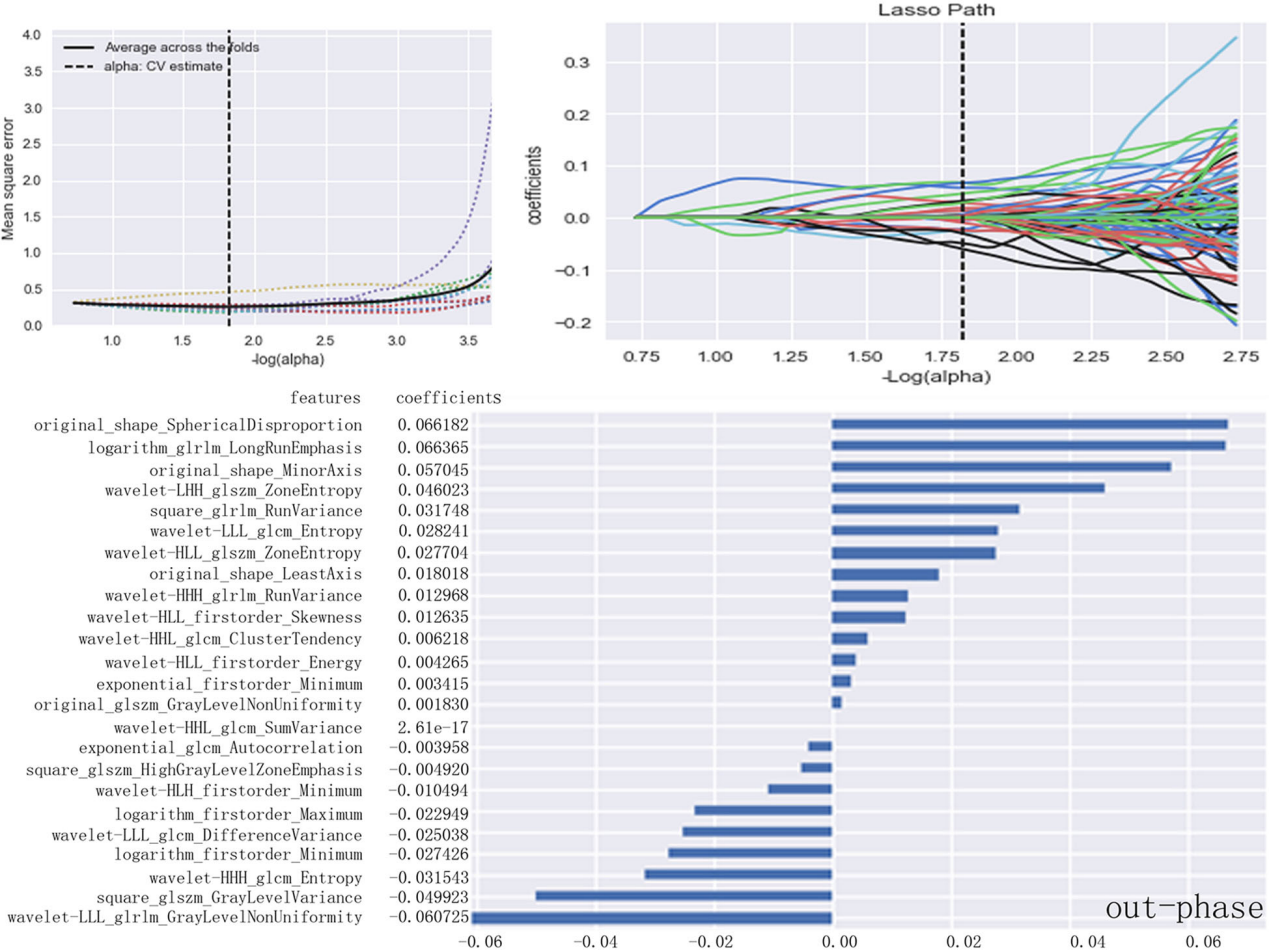
LASSO model on out-phase images. The optimal value of the lasso tuning parameter (alpha =1.823) is found. And 24 features which are correspond to the optimal alpha value are extracted following coefficients on out-phase images

**Fig. 5:**
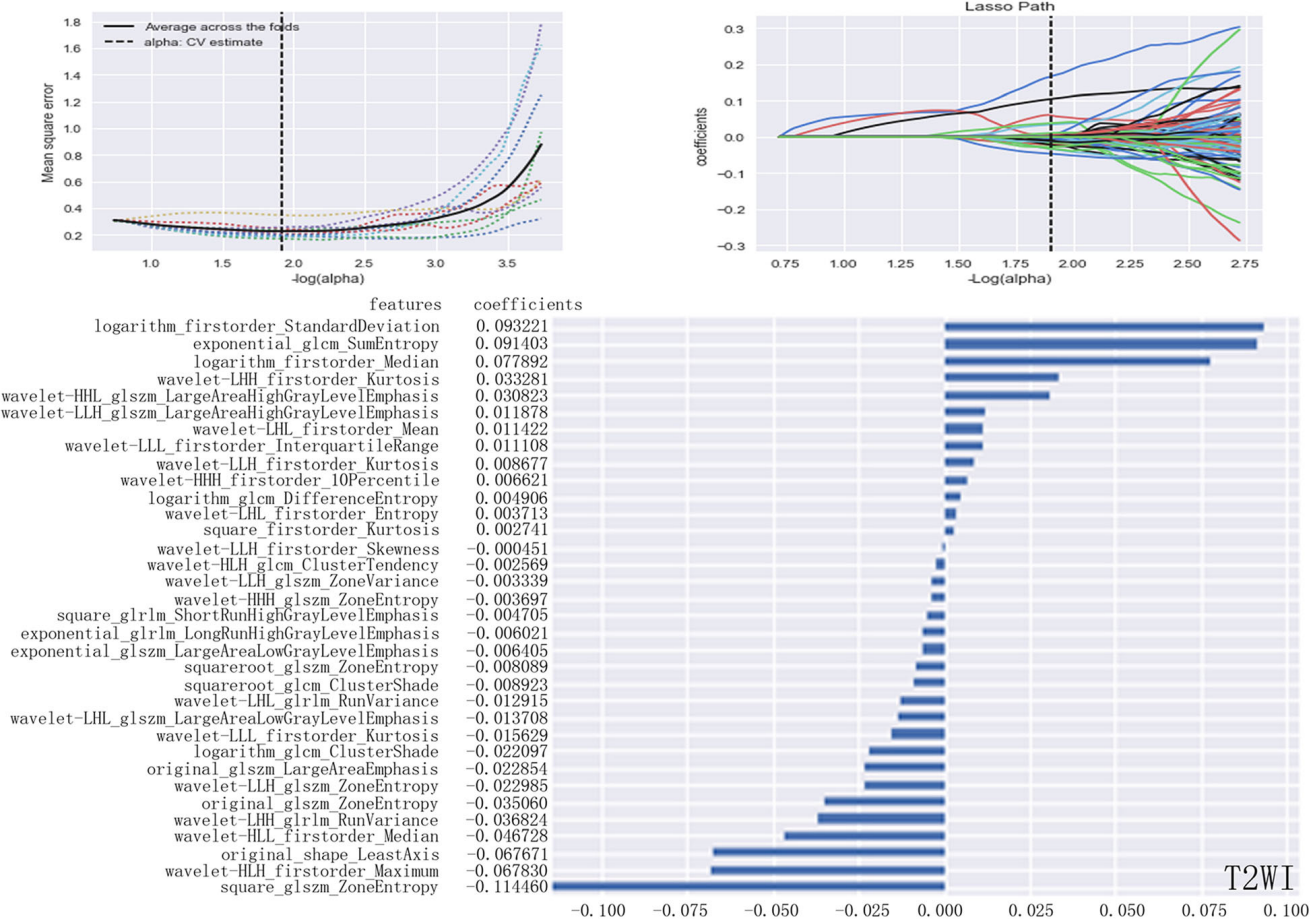
LASSO model on T2WI images. The optimal value of the lasso tuning parameter (alpha = 1.920) is found. And 34 features which are correspond to the optimal alpha value are extracted following coefficients on T2WI images

**Fig. 6:**
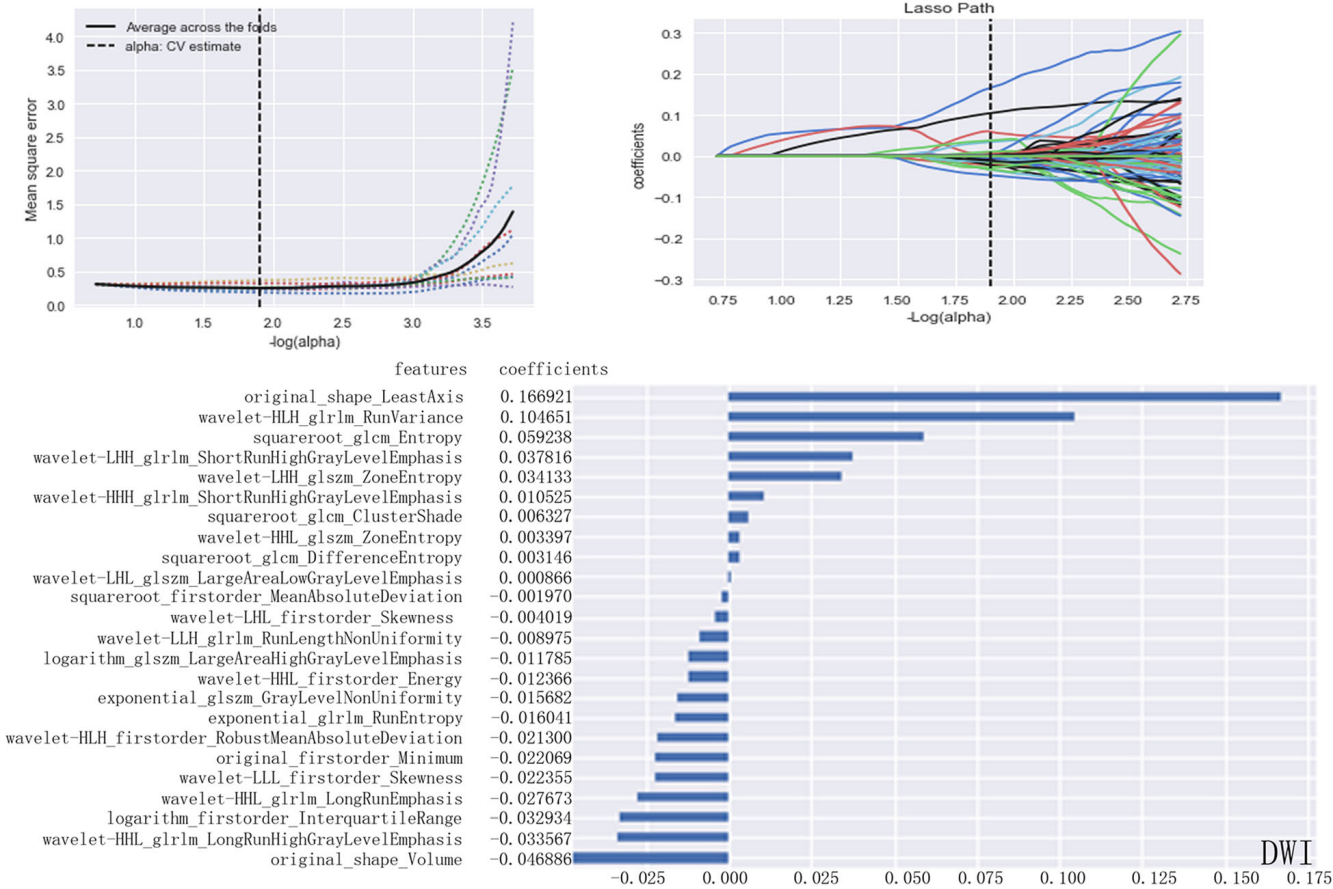
LASSO model on DWI images. The optimal value of the lasso tuning parameter (alpha = 1.903) is found. And 24 features which are correspond to the optimal alpha value are extracted following coefficients on DWI images

After calculations based on the variance threshold, 1029 basic features underwent the first dimensionality reduction on in-phase (*n* = 683), out-phase (*n* = 685), T2WI (*n* = 653), and DWI sequences (*n* = 649). Next, features with *P* values greater than 0.05 were excluded by the select k best on each sequence (in-phase: *n* = 362; out-phase: *n* = 408; T2WI: *n* = 454; DWI: *n* = 318). On the basis of these results, LASSO exported the optimal value of the LASSO tuning parameter (α), and features (in-phase: *n* = 22; out-phase: *n* = 24; T2WI: *n* = 34; DWI: n = 24) corresponding to the optimal α were derived following coefficients. A radiomics set was built using the derived features on in-phase T1WI (Fig. 3), out-phase T1WI (Fig. 4), T2WI (Fig. 5), and DWI (Fig. 6).

ROC analysis was used to evaluate the diagnostic performance of radiomics (Table 2). In comparison, the combined sequences showed optimal diagnostic performance. In the training set, the AUC was 0.86 (sensitivity: 0.76; specificity: 0.78), and in the testing set, the AUC was 0.89 (sensitivity: 0.822; specificity: 0.714).

**Table 2:** ROC analysis by the four classifiers in testing set

Decision Tree				Random Forest			
Images	AUC	Sensitivity	Specificity	Images	AUC	Sensitivity	Specificity
In-phase	0.63	0.67	0.64	In-phase	0.74	0.83	0.62
Out-phase	0.73	0.83	0.68	Out-phase	0.78	0.79	0.77
T2WI	0.68	0.72	0.68	T2WI	0.76	0.90	0.68
DWI	0.63	0.69	0.62	DWI	0.73	0.81	0.66
Combined	0.76	0.80	0.69	Combined	0.86	0.82	0.786
K Nearest Neighbours				Logistic Regression			
Images	AUC	Sensitivity	Specificity	Images	AUC	Sensitivity	Specificity
In-phase	0.66	0.73	0.57	In-phase	0.72	0.81	0.64
Out-phase	0.58	0.57	0.60	Out-phase	0.77	0.74	0.79
T2WI	0.63	0.69	0.56	T2WI	0.80	0.82	0.78
DWI	0.65	0.69	0.62	DWI	0.65	0.83	0.47
Combined	0.76	0.778	0.653	Combined	0.89	0.822	0.714

AUC, area under receiver-operating characteristic curve; T2WI, T2-weighted imaging; DWI, diffusion-weighted imaging

We summarized the diagnostic performance of single sequence where the AUC was greater than or equal to 0.75 and found that when the random forest classifier was used for classification and training with features in T2WI, the AUC was 0.76 (95% CI: 0.72–0.85; sensitivity, 0.90; and specificity, 0.68). For out-phase images, the random forest classifier yielded an AUC of 0.78 (95% CI: 0.70–0.85; sensitivity, 0.79; and specificity, 0.77). With logistic regression, the AUC in T2WI was 0.80 (95% CI: 0.74–0.80; sensitivity, 0.82; and specificity, 0.78) and that in out-phase images was 0.77 (95% CI: 0.70–0.84; sensitivity, 0.74; and specificity, 0.79). For in-phase, out-phase, T2W and DW images, classifications by the decision tree or K nearest neighbours yielded AUCs less than 0.75. When combining the four sequences’ diagnostic model, logistic regression showed better diagnostic performance with AUC of 0.89 (sensitivity, 0.822; and specificity, 0.714).

During the conventional qualitative analysis for classification of HCC and HH, the radiologist 1 (with 2 years of experience) achieved the diagnostic performance with AUC of 0.702 (95% CI: 0.65–0.75; sensitivity, 0.625; and specificity, 0.779). The radiologist 2 (with 10 years of experience) achieved the diagnostic performance with AUC of 0.908 (95% CI: 0.88–0.94; sensitivity, 0.915; and specificity, 0.901). The AUC value for radiologist 1 was significantly lower (AUC = 0.702) than that for radiologist 2 and the optimal radiomics-based combined model (AUC = 0.908, and 0.89, respectively; *p* < 0.05). Furthermore, the AUC for radiologist 2 and the optimal radiomics-based combined model had no statistic difference (p>0.05).

## Discussion

With the recent developments in precise medicine, identification of new quantitative and radiomics-based noninvasive imaging biomarkers has become a new research hotspot in radiological research [15]. The aim of our study was to develop a radiomics based system to quantitatively distinguish HCC and HH using precontrast MR-based radiomics signatures. The diagnostic power derived from various sequences and classifiers was further calculated and compared. Our results revealed that radiomics signatures could distinguish HCC and HH. The combination of four sequences with logistic regression showed improved diagnostic performance, and its AUC value (AUC = 0.89) was significantly higher than that for the less experienced radiologist (2-years experience) (AUC = 0.702). The diagnostic performances were almost equal between the radiomics-based combined model and the experienced radiologist (10-years experience) (AUC = 0.89, and 0.908, respectively; p>0.05).

The first step in building the radiomics signatures involves imaging segmentation, which may be automatically or manually performed. Here, we reviewed the detection and segmentation of abdominal organs or lesions. In order to distinguish each organ and lesion from surrounding tissues, most computers auto-recognize the edge of the target by three-dimensional space technology and simultaneously combine the shape and size to obtain the targets’ location, which is equivalent to the atlas- and landmark-based processes [22–24]. While automatic segmentation methods may greatly facilitate lesion segmentation, hepatic lesions may be associated with peripheral pathological damage, atypical shapes, and indistinct borders, which may reduce the accuracy and reproducibility of automatic segmentation [25]. There is a widespread consensus among several scholars that manual input may achieve higher reproducibility [26]. Since segmentation is critical and directly affects the subsequent processes, we were more inclined to obtain a manual sketch of lesions by two experienced radiologists.

Next, extraction of multiple features that can quantitatively represent the lesion information is of importance, which is also the core principle of radiomics [27]. Feature extraction refers to the selection of a feature subset containing the most effective features from all samples, which simplifies the classifier and also prevents the model from over-fitting. Feature extraction plays a significant role in data mining and analysis. With the explosive growth of data in recent years, large-scale and high-dimensional data have become the primary objects in computing. Gradually improving the quality of data, speeding up data analysis, and removing irrelevant and redundant features from big data have become the hotspots in data research [28, 29]. Thus, simplifying the data generation process, making models more consistent with most of the data, and reducing the possibility of over-fitting have been common goals for experts. In this study, by decreasing the dimensionality from high-throughput radiomic features, we obtained an optimal feature set which may more closely reflect the information for HCC and HH lesions. Analysis of these features showed that the shape features were the most inefficient, with only two out of 22 features on in-phase images, three out of 24 features on out-phase images, one out of 34 features on T2WI, and two out of 24 features on DWI being extracted. At present, no researcher considers shape as a reliable basis for identification. We considered that shape and volume may change over different stages during disease progression. In contrast, the higher-order statistic features occupy a significant position and may provide more valuable information for images.

Next, we elaborated on the feature set in T2WI, which showed the best performance by classification with logistic regression on single sequence. Combining the physical interpretations of radiomic features, we obtained some valuable information. For example, the uncertainty / randomness (*entropy*, *GLCM – Difference entropy*), flatness (*kurtosis*), asymmetry (*skewness*, *GLCM – cluster shade*), variation (*standard deviation*, *GLRLM – run variance*), groupings of voxels with similar greylevels (*GLCM – cluster tendency*), heterogeneity(*GLSZM – zone entropy*) in ROIs were potential identification sources in radiomics. Moreover, some of these findings were consistent with the findings of previous similar research [30]. These results maybe strongly related to the discrepant microscopic features of HCC and HH. The HH was more inclined to be uniform because of the multiple vascular channels present in it, and HCC was nonuniform with cytological atypia and heterogeneity of tumour cells.

In order to obtain the best model based on the feature set, we analysed four classifiers on in-phase, out-phase, T2WI and DWI sequences respectively. We found AUC greater than 0.75 in the T2W and out-phase images in all models. We would like to discuss the reason why T2WI and out-phase T1WI showed better performance. Pathologically, HCC consists of hepatic cancer cells that are sometimes accompanied by bleeding, calcification, necrosis, and adipose tissue [31]. In contrast, HH contains rich sinusoids and occasionally shows scar tissue and thrombus [32]. In our study, we found the out-phase T1WI showed the performance for classification of HCC and HH, this can be explained by the presence of intralesional fat for HCC. Furthermore, we found the classification performances of in- and out-phase images were different, which may result from the different signal of fat tissue on in- and out-phase images (the signal of tissue containing fat is significantly attenuated on out-phase images, and is not attenuated on in-phase images), and the random errors such as measuring, segmentation, and ROI drawing may also cause differences between in- and out-phase images. T2WI shows a decreased signal for the lipid-containing part by using fat suppression technology and a higher signal for necrosis. Furthermore, with the echo time extension, HH signals on T2WI will be higher since the sinusoids of HH are full of blood. DWI, a functional imaging technology, can noninvasively detect the Brownian motion of water molecules in living tissue. Since the DWI signal is affected by the T2 relaxation time, the degree of diffusion and differential diagnosis are mostly dependent on the apparent diffusion coefficient value [33, 34]. Thus, we speculate that it’s reasonable to obtain better performance on T2WI and out-phase images. Furthermore, we comprehensively analyzed the information of the four sequences (in-phase, out-phase T1WI, T2WI, DWI) and found that when the image information of all sequences is integrated, the characteristics of the lesions can be more accurately described by radiomics features, thus providing a more reliable basis for differential diagnosis. Further, we found the radiomics-based model with logistic regression showed significantly higher diagnostic performance than that for the less experienced radiologist (2-years experience), and the diagnostic performances were almost equal between the radiomics-based combined model and the experienced radiologist (10-years experience).

## Conclusions

This study collected the radiomics features set and suggested that the logistic regression classifier showed better predictive ability by combining four sequences. Thus, radiomics-based assessments could be used to distinguish between HCC and HH on precontrast images, thereby allowing noninvasively efficient identification and minimizing errors from visual inspection. The notable findings of our study can be summarized as follows: (1) we assessed various radiomics features including first-, second-, and higher-order statistics and shape-related features. (2) we analysed the frequently used precontrast MRI sequences in clinical practice. For these sequences, both intra- and inter-group performances were compared, which yielded an optimal model. (3) the radiomics features were developed to identify HCC and HH without invasive methods. (4) the diagnostic performance for the radiomics-based combined model with logistic regression outperformed the less experienced radiologist (2-years experience), and was nearly equal to the experienced radiologist (10-years experience).However, there are still several limitations in the radiomics research field, including the insufficient number of patients, and further studies should be performed to optimize the performance of the predictor and verify its utility.

## Abbreviations

AUC: Area under receiver-operating characteristic curve
CT: Computed tomography
DWI: Diffusion-weighted imaging
FOV: Field of view
GLCM: Gray Level Co-occurence Matrix
GLRLM: Gray Level Run Length Matrix
GLSZM: Gray Level Size Zone Matrix
HCC: Hepatocellular carcinoma
HH: Hepatic hemangioma
LASSO: Least absolute shrinkage and selection operator
MRI: Magnetic resonance imaging
NEX: Number of excitations
ROC: Receiver operating characteristic curve
ROI: Region of interest
T1WI: T1 weighted imaging
T2WI: T2 weighted imaging
TE: Echo time
TR: Repetition time

## References

[1] Omata M, Cheng AL, Kokudo N (2017). Asia-Pacific clinical practice guidelines on the management of hepatocellular carcinoma: a 2017 update. Hepatol Int.

[2] Choi BY, Nguyen MH (2005). The diagnosis and management of benign hepatic tumors. J Clin Gastroenterol.

[3] Unal E, Francis F, Aquino A, Xu R, Morgan G, Teperman L (2011). Liver transplant for mixed capillary-cavernous hemangioma masquerading as hepatocellular carcinoma in a patient with hepatocellular carcinoma. Experimental & Clinical Transplantation Official Journal of the Middle East Society for Organ Transplantation.

[4] Ghouri YA, Mian I, Rowe JH (2017). Review of hepatocellular carcinoma: epidemiology, etiology, and carcinogenesis. J Carcinog.

[5] Donato H, França M, Candelária I, Caseiro-Alves F (2017). Liver MRI: From basic protocol to advanced techniques. Eur J Radiol.

[6] Zhao W, Li W, Yi X (2016). Diagnostic value of liver imaging reporting and data system MRI on primary hepatocellular carcinoma. Zhong Nan Da Xue Xue Bao Yi Xue Ban.

[7] Galia M, Taibbi A, Marin D (2014). Focal lesions in cirrhotic liver: what else beyond hepatocellular carcinoma?. Diagn Interv Radiol.

[8] van Ginneken B (2017). Fifty years of computer analysis in chest imaging: rule-based, machine learning, deep learning. Radiol Phys Technol.

[9] Kohli M, Prevedello LM, Filice RW, Geis JR (2017). Implementing machine learning in radiology practice and research. Am J Roentgenol.

[10] Isoda T, BaBa S, Maruoka Y (2017). Influence of the different primary cancers and different types of bone metastasis on the lesion-based artificial neural network value calculated by a computer-aided diagnostic system, BONENAVI, on bone scintigraphy images. Asia Oceania journal of nuclear medicine & biology.

[11] van Ginneken B, Hogeweg L, Prokop M (2009). Computer-aided diagnosis in chest radiography: beyond nodules. Eur J Radiol.

[12] Kim Y, Furlan A, Borhani AA, Bae KT. Computer-aided diagnosis program for classifying the risk of hepatocellular carcinoma on MR images following liver imaging reporting and data system (LI-RADS). J Magn Reson Imaging. 2018;47(3):710–22. 10.1002/jmri.25772 28556283

[13] Gatos I, Tsantis S, Karamesini M (2017). Focal liver lesions segmentation and classification in nonenhanced T2-weighted MRI. Med Phys.

[14] Potter MC, Goldberg J, Aboufadel EF (1999). Advanced Engineering Mathematics[J]. Wiley.

[15] Gillies RJ, Kinahan PE, Hricak H (2016). Radiomics: images are more than pictures, They Are Data. Radiology.

[16] Haralick RM, Shanmugam K, Dinstein IH (1973). Textural Features for Image Classification. Systems Man & Cybernetics IEEE Transactions on.

[17] Krittanawong C, Zhang H, Wang Z, Aydar M, Kitai T (2017). Artificial Intelligence in Precision Cardiovascular Medicine. J Am Coll Cardiol.

[18] Venkatasubramaniam A, Wolfson J, Mitchell N, Barnes T, JaKa M, French S (2017). Decision trees in epidemiological research. Emerging themes in epidemiology.

[19] Huang L, Jin Y, Gao Y, Thung KH, Shen D (2016). Longitudinal clinical score prediction in Alzheimer’s disease with soft-Split sparse regression based random Forest. Neurobiol Aging.

[20] Jiang S, Pang G, Wu M, Kuang L (2012). An improved k-nearest neighbor algorithm for text categorization. Expert Systems with Applications An International Journal.

[21] Lei LZ, Xu YK, Hou MR, He MQ (2017). Value of PI-RADS v2 scores combined with prostate specific antigen in diagnosis of peripheral zone prostate cancer: a logistic regression analysis. Nan Fang Yi Ke Da Xue Xue Bao.

[22] Sakamoto T (2014). Roles of universal three-dimensional image analysis devices that assist surgical operations. J Hepatobiliary Pancreat Sci.

[23] Dong C, Chen YW, Foruzan AH (2015). Segmentation of liver and spleen based on computational anatomy models. Comput Biol Med.

[24] Umetsu S, Shimizu A, Watanabe H, Kobatake H, Nawano S (2014). An Automated Segmentation Algorithm for CT Volumes of Livers with Atypical Shapes and Large Pathological Lesions. Ieice Transactions on Information & Systems.

[25] Summers RM (2016). Progress in fully automated abdominal CT interpretation. Am J Roentgenol.

[26] Rios VE, Aerts HJ, Gu Y (2012). A semiautomatic CT-based ensemble segmentation of lung tumors: comparison with oncologists' delineations and with the surgical specimen. Radiother Oncol.

[27] Yip SS, Aerts HJ (2016). Applications and limitations of radiomics. Phys Med Biol.

[28] Dubey YK, Mushrif MM (2012). Extraction of wavelet based features for classification of T2-weighted MRI brain images. Signal & Image Processing.

[29] Udomhunsakul S, Wongsita P. Feature extraction in medical MRI images[P]. Cybernetics and Intelligent Systems, 2004 IEEE Conference on, 2004. 10.1109/ICCIS.2004.1460437.

[30] Zi L, Mao Y, Huang W (2017). Texture-based classification of different single liver lesion based on SPAIR T2W MRI images. Bmc Medical Imaging.

[31] Di MM, Saba L, Bosco S (2014). Hepatocellular carcinoma (HCC) in non-cirrhotic liver: clinical, radiological and pathological findings. Eur Radiol.

[32] Yong O, Cheng YD, Zhang XJ, Ouyang XH. The pathological category and the treatment of hepatic cavernous hemangiomas. Journal of Interventional Radiology. 2015;24(11):933–8.

[33] Vermoolen MA, Kwee TC, Nievelstein RAJ (2012). Apparent diffusion coefficient measurements in the differentiation between benign and malignant lesions: a systematic review. Insights Into Imaging.

[34] Lewis S, Dyvorne H, Cui Y, Taouli B (2014). Diffusion-weighted imaging of the liver : techniques and applications. Magn Reson Imaging Clin N Am.

